# Is lack of causal evidence linking socioeconomic position with health an ‘inconvenient truth’?

**DOI:** 10.1093/eurpub/ckaa004

**Published:** 2020-08-24

**Authors:** Olle Lundberg

**Affiliations:** Department of Public Health Sciences, Stockholm University, Stockholm, Sweden

Mackenbach^1^ provides a balance sheet for health inequalities research during the past decades, and suggests that our figures are in the red. Health inequalities are still present, and even growing, and even the most ambitious attempts to reduce inequalities appear to have given modest results.^2^ This development is not only due to counteracting factors in our societies, Mackenbach argues, but also attributable to the way in which mainstream thinking in the field have avoided to address and handle a set of ‘inconvenient truths’. I find this a very important discussion, and have elsewhere discussed a number of other issues that I think need reconsideration,^3^ but here I will focus on the alleged lack of evidence for a causal link between socioeconomic position (SEP) and health.

Mackenbach^1^^,^^2^ discuss the problem of causal evidence from a model including SEP, health and factors affecting both (confounders), with three types of causal relations. These are (1) from SEP to health, (2) from health to SEP and (3) from confounders to SEP and health. The argument, then, is that studies using ‘rigorous analytic methods’ have largely failed to produce much evidence for (1), while there is more evidence for (2) and, in particular, for (3).

A first critical observation is that reversed causation and confounding are not often concluded on basis of the rigorous analytic methods alluded to, but rather based on failed attempts to establish causal effects of type 1). In particular when it comes to confounding, where we assume a complex double causal relationship, ‘rigorous analytic methods’ (e.g. counterfactual approaches including quasi-experimental designs) also seem difficult to apply.

But more generally, I would argue that ‘rigorous’ methods typically conceptualize causality as a sequential relationship in strict isolation, while inequalities in conditions, opportunities and health develop through a dynamic interplay between different factors over the life-course. In addition, counterfactual approaches to causality also rests on external manipulation as a key requirement, which per definition excludes the fact that people act and react to their circumstances.^4^ Taken together, more ‘rigorous’ methods may well produce weak results not because there are no causal relations, but because they are designed to exclude the dynamic interplays that we ought to study.

Instead, inequalities in health can be viewed as created through a ‘generative process’^4^ along the life-course. This will allow a dynamic interplay between different social determinants and health statuses, where the relationship can be ‘causal’ during one phase and ‘selective’ during next. It will also make it easier to see that the outcome of one phase is the input of next, which suggests that the distinction between equality of opportunity and equality of outcome is difficult to make. Importantly, using a generative process approach will make it easier to understand the production of inequalities as an interplay between social structures, resources and human actions.

The generative process approach does not come with one specific method to apply. Rather, it rests on a need to specify theoretically the whole process, to study its different parts with methods that are applicable, and return to the bigger picture from there. To identify the sub-processes that generate inequalities, and in particular then the human action and interaction with others as well as with social structures, will be key.^4^

In order to move forward it is therefore necessary to improve our theoretical understanding of the larger process and its components. It is also important to accept that one type of study, or more generally statistical analysis on its own, can never solve how inequalities are shaped and sustained. Taking education and health as an example, there is a range of relationships that need to be considered. Education is likely to affect neuro-cognitive development as well as providing knowledge and skills (effect a). This is likely to affect conditions and opportunities in life (effect b), which in turn will affect health, but also affect health directly (effect c) as well as modify the health consequences of different conditions and opportunities (effect d). Finally, education, and in particular early education, can compensate for different childhood conditions (effect e). All these relationships (a–e) have been studied by different academic disciplines, using different methods of analysis, while the bigger issue of to what extent all these parts taken together constitute a generative process is an issue that we in the health inequalities field need to address.

In short, therefore, the ‘inconvenient truth’ is not that we lack causal evidence, but that our theoretical understanding of the bigger process that generate health inequalities need to be developed. In line with systems biology, we need to focus more on investigating and understanding complex phenomena arising from the dynamic interaction of many factors.^5^

*Conflicts of interest*: None declared. 
